# Multiple regulatory roles of AP2/ERF transcription factor in angiosperm

**DOI:** 10.1186/s40529-016-0159-1

**Published:** 2017-01-03

**Authors:** Chao Gu, Zhi-Hua Guo, Ping-Ping Hao, Guo-Ming Wang, Zi-Ming Jin, Shao-Ling Zhang

**Affiliations:** State Key Laboratory of Crop Genetics and Germplasm Enhancement, Center of Pear Engineering Technology Research, Nanjing Agricultural University, Nanjing, 210095 China

**Keywords:** AP2/ERF TF, Stress, Plant growth and development, Fruit ripening, Phytohormones

## Abstract

APETALA2/ethylene response factor (AP2/ERF) transcription factor (TF) is a superfamily in plant kingdom, which has been reported to be involved in regulation of plant growth and development, fruit ripening, defense response, and metabolism. As the final response gene in ethylene signaling pathway, AP2/ERF TF could feedback modulate phytohormone biosynthesis, including ethylene, cytokinin, gibberellin, and abscisic acid. Moreover, AP2/ERF TF also participates in response to the signals of auxin, cytokinin, abscisic acid, and jasmonate. Thus, this superfamily is key regulator for connecting the phytohormonal signals. In this review, based on the evidence of structural and functional studies, we discussed the multiple regulator roles of AP2/ERF TF in angiosperm, and then constructed the network model of AP2/ERF TF in response to various phytohormonal signals and regulatory mechanism of the cross-talk.

## Background

The surperfamily APELATA 2/ethylene response factor (AP2/ERF) has been studies in many plants, which have a range of 119–200 members (Du et al. 2014; Nakano et al. 2006; Rao et al. 2015; Zhuang et al. 2008), and have been reported in responses to ethylene, stress, metabolic, fruit ripening and senescence (Han et al. 2016; Koyama et al. 2013; Lee et al. 2012; Li et al. 2007; Fits and Memelink 2000; Trujillo et al. 2008; Zhu et al. 2014). All the time, regulatory mechanism of AP2/ERF TF in these fields were wide-spread studies by many scientists and their research teams, and increasing experimental evidence was exploited to elucidate the detailed roles in each field (Guo and Ecker 2004; Liu et al. 2014; Pré et al. 2008; Taketa et al. 2008; Tang et al. 2016; Xiao et al. 2013; Yin et al. 2016). Herein, research advance of AP2/ERF TF was reviewed in plant, and the doubtful viewpoints were also discussed.

## Classification and DNA-binding elements

According to previous reports, the superfamily AP2/ERF members contain a common DNA binding domain, AP2 domain. Based on the difference of this domain in copy numbers, AP2/ERF TF could usually be divided into four families, AP2, ERF, RAV, and Soloist (Nakano et al. 2006; Licausi et al. 2010a). AP2 members constitute by one or additionally taking a tandem repeated AP2 domain (Kagaya et al. 1999; Licausi et al. 2013). ERF members characterize by a single AP2 domain (Nakano et al. 2006; Licausi et al. 2013). RAV members comprise by a consensus sequence elements for both AP2 domain and B3 domains (Kagaya et al. 1999; Swaminathan et al. 2008). Soloist family have little members (one or two) that also contain a single AP2 domain in all sequenced plant genome, but they strongly diverged in gene sequence from other AP2/ERF members (Du et al. 2013; Licausi et al. 2010a; Rao et al. 2015; Zhuang et al. 2008). Because of ERF family members could bind to two mainly DNA-binding elements (Hao et al. 2002; Sakuma et al. 2002), resulting in a novel DREB family is separated from ERF family (Du et al. 2013; Rao et al. 2015; Sakuma et al. 2002; Zhuang et al. 2008). Of the DREB and ERF families, all the members are further classified into six groups, A1 to A6 and B1 to B6, respectively (Sakuma et al. 2002). However, these twelve groups are re-designated with group I to X, VI-L, and Xb-L or group A to J (Nakano et al. 2006). The re-designated classification is employed in horticultural plants, such as *Vitis vinifera*, *Prunus mume*, and *Solanum lycopersicon* (Licausi et al. 2010a; Du et al. 2013; Pirrello et al. 2012), whereas the traditionally classification is used in other plant species, including *Salix arbutifolia*, *Nicotiana tabacum*, and *Populus trichocarpa* (Rao et al. 2015; Sasaki et al. 2007; Zhuang et al. 2008).

AP2/ERF proteins have strongly capacity to bind a wide range of cis-regulatory elements in promoter of target genes (Sasaki et al. 2007). Of these cis-regulatory elements, GCC-box (AGCCGCC element) and DRE/CRT (dehydrationresponsive element/C-repeat, RCCGCC element) are the mainly two DNA-binding elements (De Boer et al. 2011; Fujimoto et al. 2000; Hao et al. 1998, 2002; Oñate-Sánchez et al. 2007; Wang et al. 2012). Noteworthy, most AP2/ERF proteins can bind GCC-box containing promoter, but the activation degree is different among members in various groups. For instance, the members are weak activators in group A, B and E, neutral in class G and H, and strong in group C, whereas that are as repressor in group F (Pirrello et al. 2012). Besides GCC-box and DRE/CRT, the elements diverged from these two also belong to cis-regulatory elements, which may be in response to different stimuli underlying various stresses (Mizoi et al. 2012; Shaikhali et al. 2008; Welsch et al. 2007). Moreover, ERF protein can also bind to VWRE (vascular wounding responsive element, GAAAAGAAAATTTC) and CE1 (coupling element, CACCG) in tobacco (Sasaki et al. 2007; Wu et al. 2008). In addition, few reports reveal that ERF proteins could interact directly with a non-GCC element containing promoters (Chakravarthy et al. 2003).

## Ethylene response

Ethylene is an important phytohorome for plant growth, development, senescence, and stress tolerance. Ethylene is synthesized by ACS (1-aminocyclopropane-1- carboxyla synthase) catalyzing substrate of SAM (S-adenosyl methionine) to form ACC (1-aminocyclopropane-1-carboxyla acid), and then impel by ACO (1-aminocyclopropane-1-carboxyla oxidase). Sequentially, how much ethylene produced in plant tissues are positive correlated to ACS and ACO activities. The produced ethylene in plant tissues is combined with ETR (Ethylene receptor) to activate constitutive triple response (CTR), and then induce expression of a set of ethylene insensitive (EIN) and Ethylene insensitive-like (EIL). The EIN/EIL proteins bind to upstream regions of ERF TFs to promote it expressed in tissues (Alexander and Grierson 2002; Guo and Ecker 2003; Solano et al. 1998). However, due to GCC-box usually presented in the promoter of ACS and ACO in many plants, the expressed *ERF* genes will enhance the activities of the two genes, thereby accelerate ethylene biosynthesis and signal transduction, such as *LeERF1*, *AtERF73/HRE1*, *TERF2/LeERF2*, and *MaERF9* (Li et al. 2007; Xiao et al. 2013; Yang et al. 2011; Zhang et al. 2009). Besides the positive feedback genes, few ERF TFs also represent as repressor of ACS and ACO activities to prevent ethylene biosynthesis, including *AtERF4*, *AtERF11*, *SlERF6*, and *MaERF11* (Lee et al. 2012; Li et al. 2011; Xiao et al. 2013; Yang et al. 2005). In addition, ERF.B3 has the ability to modulate the transcription levels of a subset of other ERF TFs (Liu et al. 2013). Noteworthy, this subset contains the aforementioned activators and repressors of ethylene biosynthesis and signal pathway genes. Thus, AP2/ERF TF is not only in response to ethylene signal transduction, but also can feedback regulate ethylene synthesis in plant tissues.

## Stress tolerance

Stresses are the negative environment factors around plant growth and development. Both abiotic and biotic stresses are mediated by multiple transcriptional factors, such as NAC, WRKY, MYB, bHLH, bZIP, and ERF (Abe et al. 2003; Li et al. 2013; Puranik et al. 2012; Rushton et al. 2010; Singh et al. 2002; Zhang et al. 2012a). Most studies have found the importance of AP2/ERF TF in defense of various stresses. In general, the AP2/ERF TFs in response to abiotic stresses are the members of DREB family (Licausi et al. 2013; Sakuma et al. 2002). Such as *AtERF98*, *MsERF8*, *JcERF011*, and *CaERFLP1* that enhance tolerance to salt (Chen et al. 2012; Lee et al. 2004; Tang et al. 2016; Zhang et al. 2004, 2012b). *TERF2/LeERF2*, *CBF1*, and *CBF3* exalt cold and freezing tolerances (Novillo et al. 2007; Tian et al. 2011; Zhang et al. 2010b). *Sub1A*, *SNORKEL1* and *SNORKEL2* allow rice to adapt to deep water (Fukao et al. 2006, 2011; Hattori et al. 2009; Xu et al. 2006). *HRE1* and *HRE2* improve the tolerance of the plant to the hypoxia stress (Licausi et al. 2010b). *OsWR1*, *JERF1*, *TERF1*, and *SHINE* are positive regulators of resistance to drought (Aharoni et al. 2004; Wang et al. 2012; Zhang et al. 2005, 2010a). Moreover, few of AP2/ERF TFs are involved to modulate at least two different abiotic stresses in defense response. For example, over-expression of *SlERF5* in transgenic tomato plants result in high tolerance to drought and salt stress (Pan et al. 2012). Over-expression of *JERF3* and *SodERF3* improve resistance to drought, osmotic, salt, and freezing stresses in transgenic rice and tobacco (Trujillo et al. 2008; Wu et al. 2008; Zhang et al. 2010c). Ectopic expression of *DREB2A* in *Arabidopsis* increase endurance to drought, stress, and heat stresses (Sakuma et al. 2006a, b).

Unless enhanced tolerance to abiotic stresses, AP2/ERF TF also are reported to be concerned in raising resistance to biotic stresses. Over-expression of *NtERF5* contributes to high tolerance to *Tobacco mosaic virus* in *Nicotiana tabacum* (Fischer and Droge-Laser 2004). Silence-expression of *ORA59* or *RAP2.2* results in low tolerance to *Botrytis cinerea* in *Arabidopsis thaliana* (Pré et al. 2008; Zhao et al. 2012). Loss-of-function mutants of *AtERF2* or *AtERF14* are more susceptible against *Fusarium oxysporum* in *Arabidopsis thaliana* (McGrath et al. 2005; Oñate-Sánchez et al. 2007). Exceptionally, *AtERF4* is the negatively genes in regulating *Fusarium oxysporum* resistance (McGrath et al. 2005). Similar to that in abiotic stress defense, few of AP2/ERF TFs have the ability to coordinate two or more biotic stresses in defense response. For instance, Over-expression of *MtERF1*-*1* improves tolerance to *Rhizoctonia solani* and *Phytophthora medicaginis* in *Medicago* roots (Anderson et al. 2010). Over-expression of *ERF1* in *Arabidopsis* conferred resistance to necrotrophic fungi including *B. cinerea* and *Plectosphaerella cucumerina* (Berrocal-Lobo et al. 2002). The tomato Transcription Factor *Pti4* Regulates Defense-Related Gene Expression for *Pseudomonas syringae* and *Erysiphe orontii* by combined to GCC Box and Non-GCC Box cis Elements (Chakravarthy et al. 2003).

In addition, few AP2/ERF TFs had been reported responsible for biotic and abiotic stress, simultaneously. A typical example is the positively regulator *TaPIE1* that raise the defense responses to *R. cerealis* and freezing stresses by activating defense- and stress-related genes (Zhu et al. 2014). Taken together, AP2/ERF TF plays very important roles in regulating defense response to all kinds of biotic and abiotic stresses.

## Plant growth, development, and senescence

The life of plant is cycled through seed germination, seedling growth, organ development, and senescence. In this cycle, AP2/ERF TF also displays their regulatory roles for shaping many architectural traits. In the process of seed germination, *SlERF2* positively improve transcription level of marker gene, mannanase 2, resulting in a stimulation of premature germination, and enhance hook formation of darkgrown (Pirrello et al. 2006). In the progression of plant growth and development, *AINTEGUMENTA* and *AINTEGUMENTA*-*LIKE6* are related to flower organ growth and ovule development in *Arabidopsis* (Elliott et al. 1996; Jofuku et al. 1994; Klucher et al. 1996; Krizek 2009; Mizukami and Fischer 2000). Rice ethylene-response AP2/ERF factor *OsEATB* restricts internode elongation by down-regulating ent-kaurene synthase A, leading to a reduction of rice plant height and panicle length at maturity (Qi et al. 2011). In contrast, *AtERF1*, *AtDREB1*, and *TINYT* present their ability in dwarfing plant height (Liu et al. 1998; Solano et al. 1998; Wilson et al. 1996). Moreover, *NtERF3*, *AtERF4* and *AtERF8* had been found to be associated with plant aging (Koyama et al. 2013). Of these three genes, *AtERF4* and *AtERF8* belonged to class II ERFs in *Arabidopsis*, which can accelerate precocious leaf senescence by targeting the *EPITHIOSPECIFIER PROTEIN/EPITHIOSPECIFYING SENESCENCE REGULATOR* gene and regulating the expression of many genes related to senescence (Koyama et al. 2013). In addition, AP2/ERF TF is involved in regulating metabolite productions, such as chlorophyll, wax and cutin. The present evidences show that *CitERF13* is negative regulator for chlorophyll degradation during *Citrus* fruit degreening by directly binding to the *CitPPH* promoter and enhancing the activity of a metabolite of pheophorbide hydrolase (Yin et al. 2016). *AtWIN1*, *AtSHN*, and *HvNUD* could increase an accumulation of wax and cutin on the epidermis by regulating a lipid biosynthesis pathway (Aharoni et al. 2004; Broun et al. 2004; Taketa et al. 2008). Obviously, the functions of these AP2/ERF TFs are distinctly elucidated in these reported traits, but the regulatory roles of other members should be further explored in unknown properties in future.

## Fruit ripening

Fruit is one of important tissues in fruited plants, which harbors seed formation, development, and maturity. According to respiratory intensity during ripening, fruit is divided into climacteric and non-climacteric phenotypes. The climacteric fruit must release massive ethylene at ripening, also called ethylene-dependent fruit. On the contrary, the non-climacteric fruit is ethylene-independent. To date, ethylene-dependent fleshy-fruits are the primary materials for studying fruit ripening, such as tomato, apple, and banana. In ethylene-dependent fruits, *ERF*, as the final response gene in ethylene signaling pathway, directly regulate fruit ripening by binding to the promoters of their downstream genes, including *ACO*, *ACS*, *PG*, *EXP*, and *PSY* (Han et al. 2016; Lee et al. 2012; Liu et al. 2014). At present, *LeERF1*, *MaERF9*, *MdERF1*, and *MdERF3* has been reported as the positive activator (Li et al. 2007, 2016; Wang et al. 2007; Xiao et al. 2013), whereas *SlERF6*, *MaERF11*, and *MdERF2* are the negative repressors for fruit ripening (Han et al. 2016; Lee et al. 2012). Of these *ERFs*, *MaERF9* and *MaERF11* could not only regulate the transcription levels of *ACO1* and *ACS1* by binding to their promoter, but also physically interacted with ACO1 (Xiao et al. 2013). Interestingly, MaERF11 also interact with MaHDA1, the complex repress expression levels of downstream genes targeted by MaERF11 via histone deacetylation (Han et al. 2016). Moreover, the regulatory route of *ERF* genes is intricate during fruit ripening. In apple, *MdERF2* presents at least three roads in regulating *MdACS* expression. *MdERF2* repressor and *MdERF3* activator could regulate the transcription level of *MdACS* by binding to their promoter, respectively. Meanwhile, *MdERF2* inhibit *MdERF3* activity by combining to the DRE element in the promoter, indirectly suppressed the expression level of *MdACS*. Thirdly, a directly interaction between *MdERF2* and *MdERF3* restrain the binding of *MdERF3* to the *MdACS* promoter, and then suppress the *MdACS* expressed in fruit flesh (Li et al. 2016). In tomato, *SlERF.B3* has the ability to activate the regulatory network for fruit ripening. A dominant repressor version of *SlERF.B3* (*SlERF.B3*-*SRDX*) down-regulates ethylene receptor levels, but enhances triple response and up-regulated the expression levels of *EIN3*-*like* gene, contributing to an acceleration of fruit ripening (Liu et al. 2013). Further study found that *SlERF.B3*-*SRDX* could alter the expression pattern of other ERF family members. Most notably, *SlERF.B3*-*SRDX* also stimulate the transcription levels of ripening regulators, including *RIPENING INHIBITOR* (*RIN*), *NON*-*RIPENING* (*NOR*), *COLORLESS NON*-*RIPENING* (*CNR*), and *Homeodomain*-*leucine zipper HOMEOBOX* (*HB*-*1)* (Liu et al. 2014). Therefore, the regulatory role of AP2/ERF TF is multiple, and their regulated mechanism is very complex during fruit ripening.

## Integration of phytohormonal signals

Phytohormones are a group of naturally occurring, organic substances which affected plant growth, development, and senescence at low concentrations. Of these phytohormones, auxin, cytokinin, and gibberellin are reported to be involved in regulation of seed germination and plant growth (Pacifici et al. 2015; Urbanova and Leubner-Metzger 2016; Werner et al. 2001). Ethylene plays extremely important roles in climacteric fleshy fruit ripening and senescence (Hayama et al. 2006; Xiao et al. 2013; Yin et al. 2008), and together with jasmonate and abscisic acid, participate in defense response to biotic and abiotic stresses (Li et al. 2011; Lorenzo et al. 2003; Pré et al. 2008). Obviously, cross-talk among these phytohormones must be carried out in plant tissues. This cross-talk is always surveyed by many scientists, and increasing evidences are emerged to elucidate the talk mechanism. Ethylene signal transduction is a general pathway during the life cycle of plant. As the final response gene in ethylene signaling pathway, AP2/ERF are also documented to be involved in response to other hormones. In rice, an AP2/ERF TF OsCRL5 is induced by treating with exogenous auxin, and inhibits cytokinin signal transduction by enhancing the activities of two repressors (Kitomi et al. 2011). Interestingly, several AP2/ERF TFs in subgroup B-5 are responsible for exogenous cytokinin, thereby designated as cytokinin response factor (Rashotte et al. 2006). Also in rice, *OsEATB*, which is restrained by ethylene and abscisic acid in expression level, negative regulate gibberellin biosynthesis by down-regulating a pathway gene (Qi et al. 2011). In *Arabidopsis*, tobacco and tomato, however, few AP2/ERF TFs are shown to modulate abscisic acid responses, such as *AtERF11* and *TSRF1* (Li et al. 2011; Zhang et al. 2008). The ethylene-, jasmonate-, and abscisic acid-responsive *JERF1* regulates abscisic acid biosynthesis-related gene in expression level (Zhang et al. 2004; Wu et al. 2007). Moreover, *NIC2* participate in mediating jasmonate-elicited nicotine biosynthesis (De Boer et al. 2011). *ORA59*, which was induced by jasmonate and ethylene in expression level, is the key regulator of jasmonate- and ethylene-responsive PLANT DEFENSIN 1.2 expression by binding to GCC-box element in the promoter (Pré et al. 2008; Zarei et al. 2011). *AtERF2* is a positive regulator of jasmonate-responsive defense genes, while *AtERF4* negative adjust jasmonate-responsive defense gene expression (McGrath et al. 2005). Overall, AP2/ERF TF is the key regulator to integrate all kinds of phytohormonal signals.

## Conclusions

**Fig. 1:**
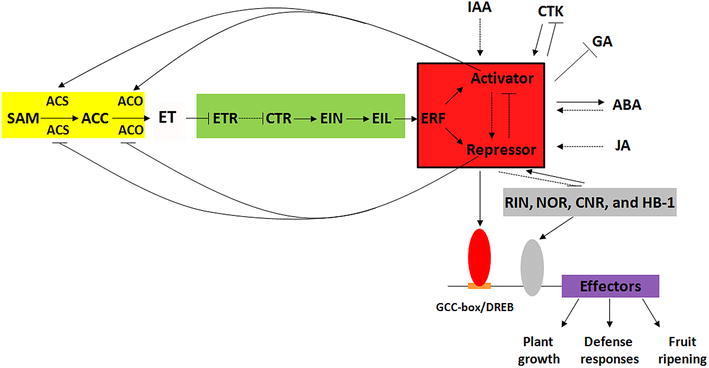
A network model for AP2/ERF genes response to phytohormones and regulating downstream effectors in angiosperm. Ethylene biosynthesis pathway, ethylene signaling pathway, and ripening regulators are indicated by *yellow*, *green*, and *gray* colors, respectively. AP2/ERF family members is *boxed* and *filled* with *red* color. In ethylene biosynthesis pathway, S-adenosine methionine (SAM) is converted to ethylene (ET) via an intermediate metabolites 1-aminocyclopropane-1-carboxyla (ACC), underlying the catalysis of the two enzymes 1-aminocyclopropane-1-carboxyla synthase (ACS) and oxidase (ACO). In ethylene signaling pathway, ET is firstly combined with ethylene receptor (ETR) to activate constitutive triple response (CTR), leading to expression of ethylene insensitive (EIN) and EIN-induced ethylene insensitive-like (EIL). EIL promote expression of ethylene response factor (ERF), including activator and repressor. The ERF activities are induced by auxin (IAA), cytokinin (CTK), abscisic acid (ABA), and jasmonate (JA), as well as ripening-related genes, such as *RIPENING INHIBITOR* (*RIN*), *NON*-*RIPENING* (*NOR*), *COLORLESS NON*-*RIPENING* (*CNR*), and Homeodomain-leucine zipper *HOMEOBOX* (*HB*-*1*). Meanwhile, ERF can reduce CTK and gibberellin (GA) levels but increase ABA biosynthesis. In ethylene responses, ERF also regulate ethylene level by enhancing and decreasing ACS/ACO activity mediated by the activators and repressors, respectively. As for effectors of plant growth, defense responses and fruit ripening, ERF can directly medium the expression by binding to GCC-box/DREB element in the promoter, and have the ability to indirectly regulate it, due to few ERFs inhibit expression of *RIN*, *NOR*, *CNR*, and *HB*-*1* that can directly bind to the promoter of effectors

The AP2/ERF superfamily has hundreds of members in various plants, which contains at least one AP2 domain in all designated families. Generally, AP2/ERF TF mediates downstream responsible genes by binding to the GCC-box and/or DREB element in the promoter. Unless responses to ethylene signal, a large number of AP2/ERF members are stimulated by auxin, cytokinin, abscisic acid, and jasmonate signals. Meanwhile, several members also modulate gibberellin, cytokinin, and abscisic acid contents by directly regulating biosynthesis pathway genes of these phytohormones. Moreover, the stimulated genes would further regulate downstream effectors, resulting in changes of agronomic traits, including plant growth, defense responses, and fruit ripening (Fig. 1). In summary, AP2/ERF TF presents multiple regulatory roles in angiosperm.
